# High-Dose versus Low-Dose Oxytocin for Labor Augmentation: A Meta-Analysis of Randomized Controlled Trials

**DOI:** 10.3390/jpm14070724

**Published:** 2024-07-04

**Authors:** Francisco Cezar Aquino de Moraes, Francinny Alves Kelly, Marianna Gerardo Hidalgo Santos Jorge Leite, Lucca Dal Moro, Victória Morbach, Rommel Mario Rodríguez Burbano

**Affiliations:** 1 Department of Medicine, Federal University of Pará, Belém 66073-005, PA, Brazil; 2 Dante Pazzanese Institute of Cardiology, São Paulo 04012-909, SP, Brazil; 3 Santa Marcelina College Medical School, São Paulo 08290-005, SP, Brazil; 4 Department of Medicine, Feevale University, Novo Hamburgo 93510-235, RS, Brazil; 5 Ophir Loyola Hospital, Belém 66063-240, PA, Brazil

**Keywords:** high-dose, low-dose, oxytocin, labor augmentation

## Abstract

**Background/Objectives:** Although oxytocin administration is recommended for delayed labor progress, there is no consensus over the preferred optimal dose of oxytocin. We aimed to perform a meta-analysis of pregnancy outcomes comparing high-dose versus low-dose oxytocin regimens for augmentation of delayed labor. **Methods:** PubMed, Embase, and Cochrane databases were systematically searched for studies comparing high-dose with low-dose oxytocin for labor augmentation from inception up to May 2023. The outcomes assessed were cesarean rate, instrumental delivery rate, postpartum hemorrhage, neonatal death, and uterine tachysystole. Subgroup analysis was performed with randomized controlled trials (RCTs) and propensity-matched studies. Statistical analysis was performed using Rstudio. Heterogeneity was assessed with I^2^ statistics, and a random-risk effect was used if I^2^ > 50%. **Results:** Twenty-one studies met inclusion criteria, and eighteen were RCTs. A total of 14.834 patients were included, of whom 7.921 (53.3%) received high-dose and 6.913 (46.6%) received low-dose oxytocin during labor augmentation. No statistical differences were found in cesarean delivery, neonatal mortality, postpartum hemorrhage and vaginal instrumentation rate. However, uterine tachysystole incidence was significantly higher with high-dose oxytocin (95% Cl, 1.30–1.94, *p* = 0.3; 0.6; I^2^ = 9%). **Conclusions:** Labor augmentation with a low-dose oxytocin regimen is effective as with a high-dose regimen, but with significantly less uterine tachysystole events, which can lead to intrauterine and neonatal complications. Our findings suggest that a low-dose regimen may be safe and effective for labor augmentation in medical practice.

## 1. Introduction

Prolonged labor is a frequent obstetric complication associated with maternal and perinatal morbidity and mortality [1]. Various conditions can contribute to its development, including abnormal fetal presentation, cephalopelvic disproportion, inadequate uterine contractions, and alterations in maternal soft tissue [2]. Currently, prolonged labor represents one of the main indications for cesarean section, a widely used surgical procedure. In this context, the search for less invasive interventions becomes crucial to reduce unnecessary cesarean rates [3]. Amniotomy and oxytocin are traditional methods used to stimulate uterine contractions in cases of dysfunctional labor [4].

Oxytocin, a hormone produced in the hypothalamus, acts in the hormonal cascade of labor, promoting an increase in the frequency, duration, and intensity of uterine contractions after the spontaneous onset of labor [5]. The stimulation of this hormone occurs directly on the smooth muscle and is physiologically increased during labor and immediately after childbirth, at which time the oxytocin receptors in the myometrium are increased [6,7].

Since the 1960s, synthetic oxytocin, commonly known as pitocin, has been widely used for labor induction and management. This medication plays a crucial role in obstetric care by aiming to promote efficient uterine contractions, favorable cervical modification, and the progression of labor towards vaginal birth [8]. By mimicking the natural hormone oxytocin, pitocin stimulates the uterus, helping to initiate and regulate contractions. This can be particularly beneficial in cases where labor needs to be induced for medical reasons, such as when a pregnancy extends beyond the due date, or if there are concerns about the health of the mother or baby [8]. The use of synthetic oxytocin can also help manage labor that is progressing too slowly. In such scenarios, it can enhance the frequency and intensity of contractions, reducing the likelihood of complications and the need for cesarean delivery [9]. Pitocin is administered intravenously, allowing healthcare providers to control the dosage precisely and adjust it according to the laboring woman’s needs and responses. Despite its widespread use and benefits, the administration of synthetic oxytocin requires careful monitoring to avoid potential risks, such as uterine hyperstimulation, which can lead to fetal distress or other complications [9].

However, oxytocin administration may be associated with adverse maternal effects, such as headache, nausea, vomiting, bradycardia, tachycardia, and cardiac arrhythmias [9]. Additionally, it can induce fetal distress, potentially culminating in hypoxia, fetal or neonatal death, and hyponatremia in the newborn [10]. A recent study demonstrated an association between oxytocin administered during labor and an increased risk of postpartum hemorrhage [11].

The optimal dose of oxytocin for stimulating contractions in cases of dysfunctional labor remains a subject of debate [12]. High doses may shorten the duration of labor, but at the cost of potentially dangerous side effects. These include uterine hypertonicity (excessive muscle tone), uterine rupture, and fetal hypoxia [12]. Conversely, low doses, while seemingly safer, might not be effective in managing prolonged labor. This dilemma underlines a critical question: can we find a middle ground between achieving efficient labor progression and ensuring the safety of both mother and baby? This ongoing debate highlights the need for further research to determine the most effective and safe oxytocin dosing strategies for labor augmentation. By pinpointing the “sweet spot” between efficacy and safety, we can strive for optimal birth outcomes for both mothers and newborns.

This systematic review and meta-analysis endeavor to delve deeper into this debate. We will meticulously analyze recently published randomized controlled trials (RCTs) to assess the safety and efficacy of high- versus low-dose oxytocin protocols for labor augmentation. Our investigation will encompass not only the success rates of vaginal delivery but also delve into crucial maternal and neonatal indicators of well-being. By critically appraising the current evidence, this meta-analysis aspires to illuminate the optimal path for oxytocin dosing in labor augmentation, ultimately promoting a smoother and safer birthing experience for mothers and their newborns.

## 2. Materials and Methods

### 2.1. Protocol and Registration

This meta-analysis was performed according to the guidelines of the declaration Preferred Reporting Items for Systematic Reviews and Meta-Analysis (PRISMA) (Tables S1 and S2) and the recommendations of the Cochrane Collaboration [13]. This review was registered on the Prospective International Registry of Systematic Reviews—PROSPERO (https://www.crd.york.ac.uk/, accessed on 27 January 2024) as number CRD42024521779.

### 2.2. Eligibility Criteria

Eligibility criteria: (1) randomized controlled trials (RCTs); (2) those comparing high-dose with low-dose oxytocin for labor augmentation; (3) involving patients aged 18 years or older who were pregnant women in spontaneous labor needing oxytocin augmentation due to delayed or slow progress of labor; (4) clinical outcomes of interest. Excluded were studies with overlapping populations, those employing medications in combination with others, non-randomized clinical trials, case reports, reviews, opinion pieces, technical reports, guidelines, animal studies, and in vitro experiments.

### 2.3. Search Strategy

We conducted a systematic search of published studies on PubMed, Cochrane and Embase. Additionally, our search extended to abstracts, articles, and scientific presentations. The database searches were carried out in May 2023. For each selected database, both Medical Subject Headings (MeSH) terms and input terms were adapted according to specific syntax rules, utilizing Boolean connectors (OR, AND). To ensure the inclusion of additional studies, we evaluated the references of the included articles and systematic reviews of the literature. Furthermore, an alert was set up in each database for notifications regarding the publication of studies relevant to our search criteria.

The search strategy was executed by two authors (F.A.K. and F.C.A.M.). In pursuit of including further studies, we assessed the references and abstracts of the included articles.

Studies identified in the databases and references from articles were integrated into the reference management software (Rayyan version 1.1)^8^. Duplicates were removed through both automatic and manual screening. Titles and abstracts of the identified articles were independently reviewed by two authors (F.A.K. and F.C.A.M.), who also independently extracted data according to predefined search criteria and quality assessment protocols. In cases of discrepancy between reviewers, a third reviewer made the final decision on inclusion.

### 2.4. Data Extraction and Risk of Bias Assessment

To summarize the main findings, three authors (V.M.S., F.A.K., and M.E.C.S.) independently collated data extracted from the included articles, including authors and year, study design, characteristics of the patient sample (size, age, clinical and pathological data, and study group), assessment method, and conclusions regarding cesarean rate, instrumental delivery rate, postpartum hemorrhage, neonatal death, and uterine tachysystole.

We employed the Cochrane Collaboration tool for assessing the risk of bias in randomized trials (RoB 2) for the quality assessment of individual randomized controlled trials (RCTs) [12]. For each trial, a risk of bias score was assigned, indicating whether it was at a high, low, or unclear risk of bias across five domains: randomization process, deviations from intended interventions, missing outcomes, measurement of outcomes, and selection of reported results. The risk of bias was independently analyzed by two researchers (F.C.A.M. and F.A.K.). The discrepancy between reviewers was resolved in agreement by the two reviewers for the final decision. We present in Figure S1 a summary of the risks of bias for the studies selected for this meta-analysis.

### 2.5. Endpoints and Definitions

The primary outcomes of interest were: (1) cesarean rate, (2) instrumental delivery rate, (3) postpartum hemorrhage, (4) neonatal death, and (5) uterine tachysystole.

### 2.6. Statistical Analysis

Researchers investigated the strength of associations between interventions and outcomes using odds ratios (ORs) and reported a range of certainty with 95% confidence intervals (CIs). To identify inconsistencies across studies (heterogeneity), Cochran’s Q-test and I^2^ statistic were employed. Studies with *p*-values below 0.10 and I^2^ exceeding 25% were considered to have significant variation [14]. The Sidik–Jonkman method was used to estimate the variation between studies (tau^2^). The DerSimonian and Laird random-effects models were implemented for all endpoints. Statistical analyses were performed using R software, version 4.4.1.

## 3. Results

### 3.1. Study Selection and Baseline Characteristics

**Figure 1:**
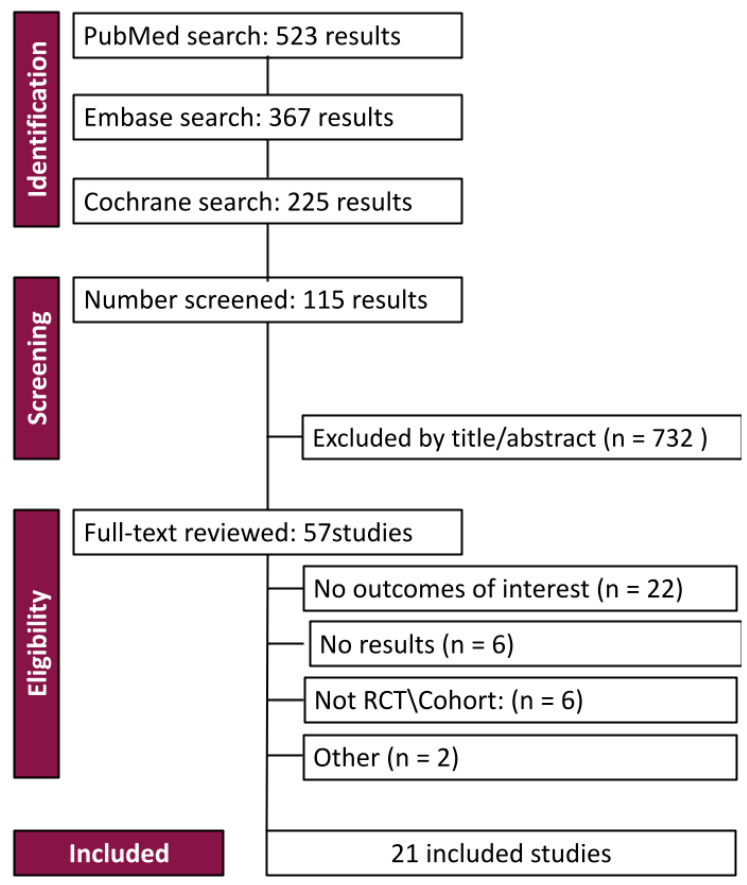
PRISMA flow diagram of study screening and selection.

In the initial literature search, 1115 results were identified. After removing duplicates and ineligible studies, twenty-one studies remained, of which eighteen were RCTs, as seen in Figure 1.

After full review according to prespecified criteria, a total of 14.834 patients were evaluated through comparison of 7.921 (53.39%) women who received high doses of oxytocin with 6.913 patients (46.6%) who received low doses for labor augmentation. The mean age was reported as 26.47 years for the high-dose group and 27.61 years for the low-dose group. Moreover, the mean gestational age presented a minimal difference of 0.12 weeks between the groups. Nulliparous women constituted the majority of the participants, with a count of 12,207, while the initial oxytocin dosage averaged at 5.05 mU/min for the intervention group and 1.82 mU/min for the control group. Study characteristics are shown in Table 1 [15,16,17,18,19,20,21,22,23,24,25,26,27,28,29,30,31,32,33,34,35]. We received no notifications in any of the databases that had set up alerts. No studies were included after the review.

### 3.2. Cesarean

**Figure 2:**
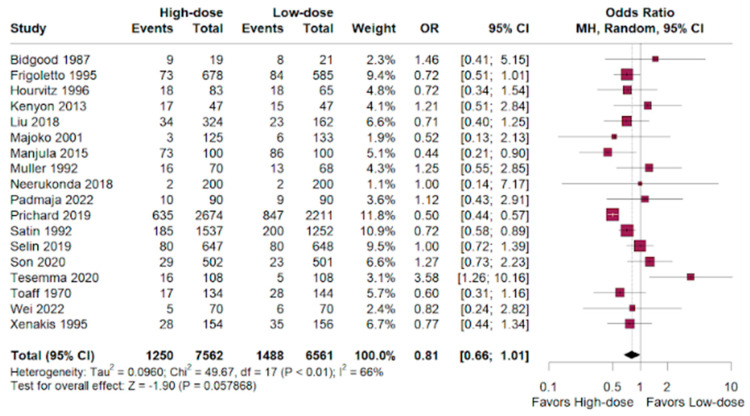
Cesarean outcomes of women who underwent labor augmentation using oxytocin, comparing high-dose versus low-dose treatments [15,16,17,18,19,20,21,22,23,24,25,26,27,28,29,30,31,32,33,34,35].

Cesarean section had a favorable outcome for the high-dosage group in patients randomized compared to the low-dose outcomes. (OR 0.81; 95% CI 0.66–1.01; *p* < 0.01; I^2^ = 66%; Figure 2).

### 3.3. Hemorrhage

**Figure 3:**
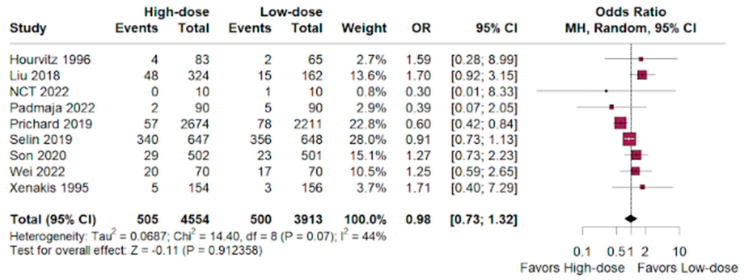
Hemorrhage outcomes of women who underwent labor augmentation using oxytocin, comparing high-dose versus low-dose treatments [15,16,17,18,19,20,21,22,23,24,25,26,27,28,29,30,31,32,33,34,35].

The hemorrhage odds ratio was nearly identical between both groups, with a slight lean towards the high-dose group versus low-dosage group. (OR 0.98; 95% CI 0.73–1.32; *p* < 0.01; I^2^ = 44%; Figure 3).

### 3.4. Neonatal Death

**Figure 4:**
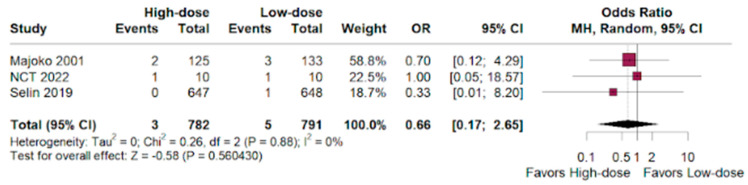
Neonatal deaths by newborns of women who underwent labor augmentation using oxytocin, comparing high-dose versus low-dose treatments [15,16,17,18,19,20,21,22,23,24,25,26,27,28,29,30,31,32,33,34,35].

The odds ratio indicated prevalence for the high-dose group over the low-dose group in women receiving labor augmentation with oxytocin. (OR 0.66; 95% CI 0.17–2.65; *p* < 0.01; I^2^ = 0%; Figure 4).

### 3.5. NICU

**Figure 5:**
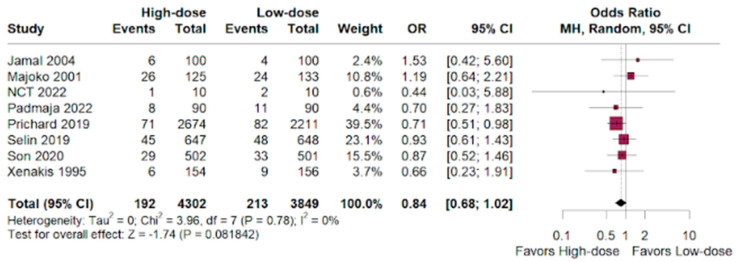
NICU admissions by newborns of women who underwent labor augmentation using oxytocin, comparing high-dose versus low-dose treatments [15,16,17,18,19,20,21,22,23,24,25,26,27,28,29,30,31,32,33,34,35].

Among NICU admissions, there was a slight benefit for the high-dose group versus the low-dose group in women who underwent labor augmentation. (OR 0.84; 95% CI 0.68–1.02; *p* < 0.01; I^2^ = 0%; Figure 5).

### 3.6. Instrumental Vaginal Delivery

**Figure 6:**
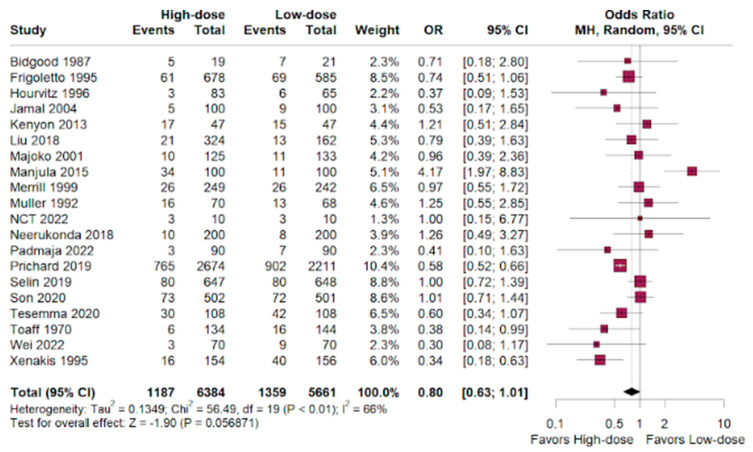
Instrumental vaginal delivery performed in women who underwent labor augmentation using oxytocin, comparing high-dose versus low-dose treatments [15,16,17,18,19,20,21,22,23,24,25,26,27,28,29,30,31,32,33,34,35].

The OR was slightly favorable for the high-dosage women over the low-dosage women, who were administered oxytocin for labor augmentation. (OR 0.80; 95% CI 0.63–1.01; *p* < 0.01; I^2^ = 66%; Figure 6).

### 3.7. Uterine Tachysystole

**Figure 7:**
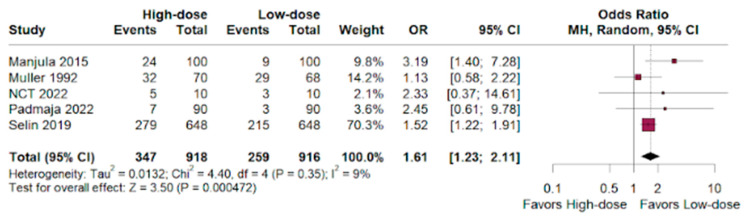
Uterine tachysystole in deliveries of women who underwent labor augmentation using oxytocin, comparing high-dose versus low-dose treatments [15,16,17,18,19,20,21,22,23,24,25,26,27,28,29,30,31,32,33,34,35].

The odds ratio for uterine tachysystoles showed a benefit for the low-dose group versus the high-dose group of women who were given oxytocin for labor augmentation (HR 1.61; 95% CI 1.23–2.11; *p* < 0.01; I^2^ = 9%; Figure 7).

## 4. Discussion

This systematic review and meta-analysis of randomized controlled trials investigated the use of high-dose versus low-dose oxytocin for labor augmentation, analyzing data from 14.834 patients across 21 studies. While the primary focus of this work was on the cesarean delivery rate, the analysis also considered other relevant outcomes such as hemorrhage, neonatal death, NICU admission, instrumental vaginal delivery, and uterine tachysystole.

As previous studies had divergent conclusions [36,37], this analysis found unsubstantial differences in the cesarean delivery rate between the high-dose and low-dose oxytocin groups (OR 0.81; 95% CI 0.66–1.01; *p* < 0.01). Hemorrhage rates were similar between groups, with a slight non-significant tendency towards the high-dose group (OR 0.98; 95% CI 0.73–1.32; *p* < 0.01). High-dose oxytocin showed a potential advantage in terms of neonatal deaths, with a lower odds ratio compared to low-dose oxytocin (OR 0.66; 95% CI 0.17–2.65; *p* < 0.01), but the confidence interval was wide and the only eight events demonstrate that these benefits are statistically insignificant and hinder the validation of the results.

A insignificant benefit was observed for high-dose oxytocin for NICU admissions (OR 0.84; 95% CI 0.68–1.02; *p* < 0.01), and we observed an inconclusive advantage for the high-dose group compared to the low-dose group in achieving instrumental vaginal delivery (OR 0.80; 95% CI 0.63–1.01; *p* < 0.01). The high-dose group had a significantly higher risk of uterine tachysystole compared to the low-dose group (HR 1.61; 95% CI 1.23–2.11; *p* < 0.01).

Thus, our meta-analysis suggests that low-dose oxytocin might be a viable alternative to high-dose for labor augmentation. While high-dose oxytocin may offer a potential benefit in terms of a shorter duration of labor, it comes with a significantly increased risk of uterine tachysystole, which can diminish fetal oxygenation by reducing placenta blood flow [38]. Other results such as NICU admissions, cesarean delivery rates, and postpartum hemorrhages (blood loss of more than 500 mL on both vaginal and cesarean deliveries) [39] showed statistically insignificance, and considering the lack of significant differences in delivery outcomes and potential adverse events, low-dose oxytocin appears to be a safer and equally effective choice [40].

This meta-analysis has some limitations. First, the heterogeneity of some outcomes may reflect the high variability in the characteristics of the patients included in this study. Second, some characteristics such as race, location, previous diseases, and BMI were not available, which may have limited the generalizability of our results to different populations. However, these limitations did not prevent robust conclusions from being drawn, and low-dose oxytocin appears to be as effective as high-dose oxytocin for labor augmentation in terms of cesarean and instrumental delivery; it is also potentially safer due to a lower risk of uterine tachysystole.

Future studies are needed to confirm our results and further explore neonatal outcomes and women’s experiences; we must also assess the influence of parity and BMI on treatment outcomes. Additionally, it is crucial to investigate the long-term effects of administering low-dose oxytocin on both mothers and infants to ensure its safety and efficacy over extended periods. Furthermore, a deeper examination of the psychological and emotional impacts on women undergoing this treatment could provide a more comprehensive understanding of their overall well-being.

Moreover, research should aim to identify any potential variations in treatment outcomes across different demographic groups, considering factors such as age, ethnicity, and socioeconomic status. These studies could help in tailoring treatment protocols to better suit diverse populations. It would also be beneficial to conduct large-scale, multi-center trials to enhance the generalizability of the findings and to solidify the evidence base supporting the use of low-dose oxytocin.

In parallel, qualitative studies focusing on women’s subjective experiences and satisfaction with low-dose oxytocin treatment could offer valuable insights into patient-centered care approaches. Understanding the preferences and expectations of women could inform the development of guidelines that align more closely with their needs and improve overall treatment experiences.

However, this paper provides valuable insights into low-dose oxytocin as a potentially safer and comparably effective alternative to high-dose oxytocin. The findings suggest that low-dose oxytocin may reduce the risk of adverse effects associated with higher doses, while still achieving desirable clinical outcomes. This represents a significant step forward in optimizing labor induction practices and enhancing maternal and neonatal health.

## 5. Conclusions

This meta-analysis of randomized controlled trials provides convincing evidence that low-dose oxytocin can be as effective as high-dose oxytocin for labor augmentation. In addition, it has proven to be safer for use in medical practice, being effective without the increased risk of uterine tachysystoles observed with the high-dose regimen, which can lead to complications even if it extends the duration of labor. Moreover, to address the limitations of heterogeneity in outcomes and high variability in patient features, further research is needed to explore the optimal dosage of oxytocin that considers individual patient’s characteristics, risk factors, neonatal outcomes, and women’s experiences. Ultimately, the choice of the ideal oxytocin dosage should be individualized through a personalized approach, considering the potential benefits and risks in each case.

## Figures and Tables

**Table 1:** Design and characteristics of studies included in the meta-analysis.

Study, Year	Design	Intervention	Control	Gestational Age	Age (Mean)	Nullipara	Cervical Dilatation
Bidgood, 1987 [15]	RCT	high dose (19)	low dose (21)	>34 weeks	NI	NI	<0.5 cm/h
Frigoletto, 1995 [16]	RCT	high dose (678)	low dose (585)	≥36 weeks	NI	I: 678 (100%) C:585 (100%)	I: 3.3 ± 2.0 C: 3.6 ± 2.1
Hourvitz, 1996 [17]	RCT	high dose (83)	low dose (65)	I: 41.0 ± 51.5 C: 40.7 ± 1.5	I: 28.4 ± 5.0 C: 28.7 ± 5.4	I: 47 (56.1%) C:34 (51.9%)	I: 1.8 ± 0.9 C: 1.7 ± 0.9
Jamal, 2004 [18]	RCT	high dose (100)	low dose (100)	I: 39.1 ± 1.3 C: 38.0 ± 1.3	I: 25.4 ± 4.9 C: 26.3 ± 5.1	I: 50 (50%) C:41 (41%)	I: 3.5 ± 0.7 C: 3.7 ± 0.7
Kenyon, 2013 [19]	RCT	high dose (47)	low dose (47)	≥37 weeks	NI	I: 47 (100%) C:47 (100%)	>4 cm
Liu, 2018 [20]	RCT	high dose (324)	low dose (162)	I: 278 ± 4 days C: 281 ± 5 days	I: 25.53 ± 2.46 C: 25.46 ± 1.95	I: 138 (85%) C:142 (88%)	>28 mm
Majoko, 2001 [21]	RCT	high dose (125)	low dose (133)	I: 39.2 ± 2.1 C: 39.7 ± 2.1	I: 20.2 ± 3.3 C: 20.4 ± 3.5	I: 125 (100%) C:133 (100%)	I: 6.0 ± 1.6 C: 6.3 ± 1.7
Manjula, 2015 [22]	RCT	high dose (100)	low dose (100)	I: 38.2 ± 1.07 C: 38.0 ± 1.11	I: 26.0 ± 3.4 C: 26.2 ± 3.7	I: 65 (65%) C:63 (63%)	≥3 cm
Merril, 1999 [23]	double-blind RCT	high dose (249)	low dose (242)	I: 39.1 ± 0.1 C: 38.9 ± 0.1	I: 25.4 ± 0.4 C: 25.4 ± 0.4	I: 135 (54.2%) C:115 (47.5%)	I: 4.7 ± 0.1 C: 5.0 ± 0.1
Muller, 1992 [24]	RCT	high dose (70)	low dose (68)	I: 40.5 ± 1.6 C: 40.6 ± 1.5	I: 22.7 ± 5.7 C: 22.3 ± 5.3	I: 46 (65.7%) C:45 (66.2%)	I: 1.7 ± 1.1 C:1.8 ± 1.1
NCT, 2022 [25]	RCT	high dose (10)	low dose (10)	38.7 ± 1.85	26.2 ± 5.26	NI	NI
Neerukonda, 2018 [26]	RCT	high dose (200)	low dose (200)	I: 38.9 ± 0.88 C: 39.0 ± 0.86	I: 24.1 ± 3.5 C: 24.4 ± 3.2	I: 98 (49%) C:98 (49%)	NI
Padmaja, 2022 [27]	RCT	high dose (90)	low dose (90)	I: 38.6 ± 2.5 C: 38.1 ± 1.2	I: 25.0 ± 4.0 C: 24.7 ± 4.1	I: 90 (100%) C:90 (100%)	≥3 cm
Prichard, 2019 [28]	retrospective	high dose (2674)	low dose (2211)	I: 39.9 ± 1.30 C: 39.7 ± 1.30	I: 30.3 ± 4.7 C: 30.7 ± 4.6	I: 2674 (100%) C:2211 (100%)	I: 645 (29.2%) C: 1016 (38%)
Selin, 2019 [29]	RCT	high dose (647)	low dose (648)	I: 29.9 ± 4.8 C: 29.9 ± 4.6	I: 29.0 ± 4.8 C: 29.0 ± 4.6	I: 647 (100%) C:648 (100%)	I: 3.47 ± 1.55 C: 3.50 ± 1.60
Satin, 1992 [30]	RCT	high dose (1537)	low dose (1252)	I: 39.0 0 ± 0.1 C: 39.0 0 ± 0.1	I: 22.7 0 ± 0.2 C: 22.9 ± 0.2	I: 938 (61%) C:751 (60%)	≥3 cm I: 48% C:47%
Son, 2020 [31]	double-blind RCT	high dose (502)	standard dose (501)	I: 39.1 ± 0.8 C: 39.1 ± 0.7	I: 31.5 ± 4.4 C: 31.7 ± 4.3	I: 502 (100%) C:501 (100%)	≥3 cm
Tesemma, 2020 [32]	cross-sectional	high dose (108)	low dose (108)	39.4 weeks	26 years	I: 56 (51.9%) C:32 (29.6%)	≥4 cm
Toaff, 1978 [33]	prospective	pharmacological dose (134)	physiological dose (144)	I: 39.51 ± 0.95 C: 43.62 ± 4.27	I: 24- 1 ± 2.5 C: 23.2 ± 2.3	I: 64 (47.74%) C:62 (43.06%)	NI
Wei, 2022 [34]	double-blind RCT	high dose (70)	low dose (70)	≥37 weeks	I: 27.67 ± 4.86 C: 29.73 ± 5.54	I: 21 (30%) C:22 (31.43%)	NI
Xenakis, 1995 [35]	RCT	high dose (154)	low dose (156)	I: 40.2 ± 1.6 C: 39.9 ± 2.0	I: 24.4 ± 5.9 C: 24.2 ± 6.9	I: 72 (46.75%) C:94 (60.26%)	I: 5.4 cm C: 6.2 cm

I: intervention; C: control; NI: not informed. Values in mean ± SD or n (%).

## Data Availability

Data sharing is not applicable to this article.

## Supplementary Materials

The following supporting information can be downloaded at: https://www.mdpi.com/article/10.3390/jpm14070724/s1. Table S1: PRISMA 2020 Checklist; Table S2: PRISMA 2020 for Abstract Checklist; Figure S1: Methodological quality summary using RoB2. A Overall risk of bias.
