# Supplementary material for: High-Dose versus Low-Dose Oxytocin for Labor Augmentation: A Meta-Analysis of Randomized Controlled Trials
Source: J Pers Med. 2024 Jul 4;14(7):724. doi: 10.3390/jpm14070724 (PMC11278403; doi:10.3390/jpm14070724)

Supplementary Material

Supplementary Table S1 PRISMA 2020 Checklist.

Supplementary Table S2 PRISMA 2020 for Abstract Checklist.

Supplementary Figure S1 Methodological quality summary using RoB2. A Overall risk of bias

Table S1. PRISMA 2020 Checklist.

Section and Topic	Item #	Checklist item	Location where item is reported
TITLE			
Title	1	**Effects of AKT inhibitors for Advanced or Metastatic Breast Cancer: A Meta-analysis Of Randomized Clinical Trials.**	Page 1
ABSTRACT			
Abstract	2	See the PRISMA 2020 for Abstracts checklist.	Supplementary Materials: Table S3.
INTRODUCTION			
Rationale	3	The efficacy of adding AKT inhibitors to the treatment of advanced or metastatic breast cancer is not yet fully elucidated. Therefore, the study aims to evaluate the benefits of using AKT inhibitors in patients with advanced or metastatic breast cancer, with emphasis on variables	Page 4
Objectives	4	Verify the efficacy of adding AKT inhibitors to the treatment for advanced or metastatic breast cancer	Page 4
METHODS			
Eligibility criteria	5	Studies that met the following eligibility criteria were included: (1) RCTs; (2) with or without AKT inhibitors; (3) patients ≥18 years of age with advanced or metastatic breast cancer that was not amenable to curative therapy; (4) patients with Eastern Cooperative Oncology Group (ECOG) performance status score of 0, 1, or 2; (5) (6) patients could have prior radiotherapy or hormone therapy (REF)	Page 5
Information sources	6	Pubmed, Cochrane Library and Embase were systematically searched	Page 5
Search strategy	7	Present the full search strategies for all databases, registers and websites, including any filters and limits used.	Page 17
Selection process	8	Specify the methods used to decide whether a study met the inclusion criteria of the review, including how many reviewers screened each record and each report retrieved, whether they worked independently, and if applicable, details of automation tools used in the process.	Page 5

Data collection process	9	Specify the methods used to collect data from reports, including how many reviewers collected data from each report, whether they worked independently, any processes for obtaining or confirming data from study investigators, and if applicable, details of automation tools used in the process.	Page 5
Data items	10a	List and define all outcomes for which data were sought. Specify whether all results that were compatible with each outcome domain in each study were sought (e.g. for all measures, time points, analyses), and if not, the methods used to decide which results to collect.	Pages 5
	10b	List and define all other variables for which data were sought (e.g. participant and intervention characteristics, funding sources). Describe any assumptions made about any missing or unclear information.	Page NO
Study risk of bias assessment	11	The Cochrane Collaboration tool for assessing risk of bias in randomized trials (RoB 2) was utilized for quality assessment of individual randomized studies(REF). Three authors (E.P., L.M.L. and F.C.A.M.) independently conducted the risk of bias assessment and disagreements were resolved by consensus. Each trial was assigned a score of high, low, or unclear risk of bias across five domains: randomization process, deviations from intended interventions, missing outcomes, measurement of outcomes, and selection of reported results. Funnel-plot analyzes were employed to examine publication bias (REF).	Page 6
Effect measures	12	Specify for each outcome the effect measure(s) (e.g. risk ratio, mean difference) used in the synthesis or presentation of results.	Page 6
Synthesis methods	13a	Describe the processes used to decide which studies were eligible for each synthesis (e.g. tabulating the study intervention characteristics and comparing against the planned groups for each synthesis (item #5)).	Pages NO
	13b	Describe any methods required to prepare the data for presentation or synthesis, such as handling of missing summary statistics, or data conversions.	Pages NO
	13c	Describe any methods used to tabulate or visually display results of individual studies and syntheses.	Page 6
	13d	Describe any methods used to synthesize results and provide a rationale for the choice(s). If meta-analysis was performed, describe the	Page 6

		model(s), method(s) to identify the presence and extent of statistical heterogeneity, and software package(s) used.	
	13e	Describe any methods used to explore possible causes of heterogeneity among study results (e.g. subgroup analysis, meta-regression).	Pages NO Just in Results Page 8
	13f	Describe any sensitivity analyses conducted to assess robustness of the synthesized results.	Pages 6
Reporting bias assessment	14	Describe any methods used to assess risk of bias due to missing results in a synthesis (arising from reporting biases).	Pages No
Certainty assessment	15	Describe any methods used to assess certainty (or confidence) in the body of evidence for an outcome.	Pages 6
RESULTS			
Study selection	16a	Describe the results of the search and selection process, from the number of records identified in the search to the number of studies included in the review, ideally using a flow diagram.	Page 7 Figure 1
	16b	Cite studies that might appear to meet the inclusion criteria, but which were excluded, and explain why they were excluded.	Supplementary Materials: Table S.
Study characteristics	17	Cite each included study and present its characteristics.	Pages 7 Table 1
Risk of bias in studies	18	Present assessments of risk of bias for each included study.	Pages 11-12
Results of individual studies	19	For all outcomes, present, for each study: (a) summary statistics for each group (where appropriate) and (b) an effect estimate and its precision (e.g. confidence/credible interval), ideally using structured tables or plots.	Pages 9-11 Figure 2, 3 and 4
Results of syntheses	20a	For each synthesis, briefly summarise the characteristics and risk of bias among contributing studies.	Pages 9-11 Figure 2, 3 and 4
	20b	Present results of all statistical syntheses conducted. If meta-analysis was done, present for each the summary estimate and its precision (e.g. confidence/credible interval) and measures of statistical heterogeneity. If comparing groups, describe the direction of the effect.	Pages 9-11 Figure 2, 3 and 4
	20c	Present results of all investigations of possible causes of heterogeneity among study results.	Page 12
	20d	Present results of all sensitivity analyses conducted to assess the robustness of the synthesized results.	Page 12
Reporting biases	21	Present assessments of risk of bias due to missing results (arising from reporting biases) for each synthesis assessed.	Page NO

Certainty of evidence	22	Present assessments of certainty (or confidence) in the body of evidence for each outcome assessed.	Page 12
DISCUSSION			
Discussion	23a	Provide a general interpretation of the results in the context of other evidence.	Pages 12-14
	23b	Discuss any limitations of the evidence included in the review.	Page 13
	23c	Discuss any limitations of the review processes used.	Page 13
	23d	Discuss implications of the results for practice, policy, and future research.	Page 14
OTHER INFORMATION			
Registration and protocol	24a	Provide registration information for the review, including register name and registration number, or state that the review was not registered.	Page 6
	24b	Indicate where the review protocol can be accessed, or state that a protocol was not prepared.	Page 6
	24c	Describe and explain any amendments to information provided at registration or in the protocol.	Page 6
Support	25	Describe sources of financial or non-financial support for the review, and the role of the funders or sponsors in the review.	Page 15
Competing interests	26	Declare any competing interests of review authors.	Page 16
Availability of data, code and other materials	27	Report which of the following are publicly available and where they can be found: template data collection forms; data extracted from included studies; data used for all analyses; analytic code; any other materials used in the review.	Page NO

Table S2. PRISMA 2020 for Abstract Checklist.

Section and Topic	Item #	Checklist item	Reported (Yes/No)
TITLE			
Title	1	Identify the report as a systematic review.	Yes
BACKGROUND			
Objectives	2	Provide an explicit statement of the main objective(s) or question(s) the review addresses.	Yes
METHODS			
Eligibility criteria	3	Specify the inclusion and exclusion criteria for the review.	No
Information sources	4	Specify the information sources (e.g. databases, registers) used to identify studies and the date when each was last searched.	Yes
Risk of bias	5	Specify the methods used to assess risk of bias in the included studies.	No
Synthesis of results	6	Specify the methods used to present and synthesise results.	No
RESULTS			
Included studies	7	Give the total number of included studies and participants and summarise relevant characteristics of studies.	Yes
Synthesis of results	8	Present results for main outcomes, preferably indicating the number of included studies and participants for each. If meta-analysis was done, report the summary estimate and confidence/credible interval. If comparing groups, indicate the direction of the effect (i.e. which group is favoured).	Yes
DISCUSSION			
Limitations of evidence	9	Provide a brief summary of the limitations of the evidence included in the review (e.g. study risk of bias, inconsistency and imprecision).	No
Interpretation	10	Provide a general interpretation of the results and important implications.	Yes
OTHER			
Funding	11	Specify the primary source of funding for the review.	Yes
Registration	12	Provide the register name and registration number.	Yes

Supplementary Figure S1. Methodological quality summary using RoB2. **A** Overall risk of bias

A. Overall risk of bias

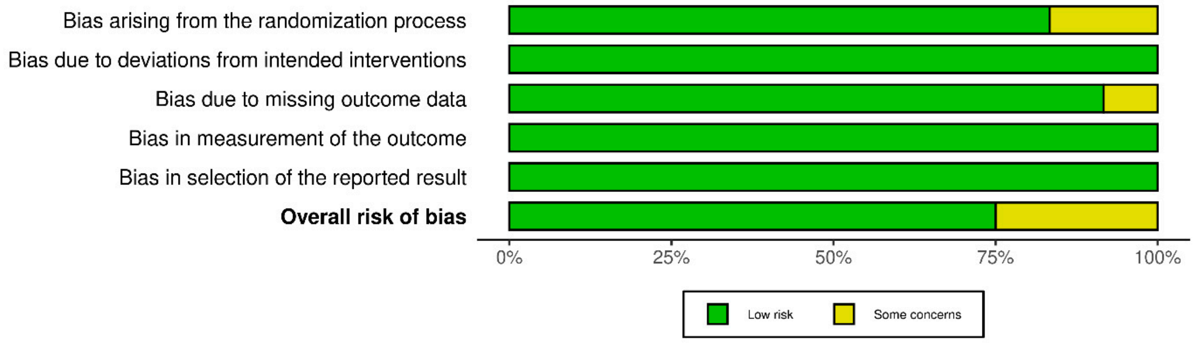

Supplement: Supplementary file 1 [file jpm-14-00724-s001.zip › jpm-2999523-supplementary.pdf]
